# Sex Differences in Cutaneous Melanoma: Incidence, Clinicopathological Profile, Survival, and Costs

**DOI:** 10.1089/jwh.2021.0223

**Published:** 2022-07-12

**Authors:** Alessandra Buja, Massimo Rugge, Giovanni Damiani, Manuel Zorzi, Chiara De Toni, Antonella Vecchiato, Paolo Del Fiore, Romina Spina, Vincenzo Baldo, Alessandra Rosalba Brazzale, Carlo Riccardo Rossi, Simone Mocellin

**Affiliations:** ^1^ Department of Cardiologic, Vascular and Thoracic Sciences, and Public Health, Health Care Services and Health Promotion Evaluation, Hygiene and Public Health Unit, University of Padua, Padua, Italy.; ^2^ Veneto Tumor Registry, Azienda Zero, Padua, Italy.; ^3^ Department of Medicine-DIMED, Pathology and Cytopathology Unit, University of Padua, Padua, Italy.; ^4^ Clinical Dermatology, IRCCS Istituto Ortopedico Galeazzi, Milan, Italy.; ^5^ Department of Biomedical, Surgical and Dental Sciences, University of Milan, Milan, Italy.; ^6^ PhD Program in Pharmacological Sciences, Department of Pharmaceutical and Pharmacological Sciences, University of Padua, Padua, Italy.; ^7^ Departament of Statistical Sciences, University of Padua, Padua, Italy.; ^8^ Soft-Tissue, Peritoneum and Melanoma Surgical Oncology Unit, IOV-IRCCS, Padua, Italy.; ^9^ Department of Surgery, Oncology and Gastroenterology (DISCOG), University of Padua, Padua, Italy.

**Keywords:** melanoma, incidence, survival, histopathological characteristics, sex disparities

## Abstract

**Background:**

This study aims to provide a comprehensive overview of sex-related characteristics of cutaneous malignant melanoma (CMM), with special reference to its incidence, clinicopathological profile, overall survival, and treatment-related costs.

**Methods:**

This retrospective cohort study included all 1,279 CMM patients who were registered in 2015 in the Veneto Cancer Registry (a population-based registry including all 4,900,000 regional residents). The by-sex comparisons included tumor stage and site, histological subtype, and other clinical–pathological variables. A Cox regression analysis was used to test the association between sex and survival, adjusting for the main covariates. Treatment costs were calculated by linking patients with several administrative regional databases.

**Results:**

Age-specific incidence rates were significantly higher for men among people >50 years old. For men, the trunk was the most common primary site (59.3%), whereas for women the lower limbs (32.1%) were the most common primary site, followed by the trunk (31.8%), which was lower than for men (*p* < 0.001). At presentation, the frequency of early stage CMM was higher among women, who also featured a significantly lower risk of death (*p* = 0.016), after adjusting for covariates. Men also incurred higher costs for melanoma treatment in the first year after their diagnosis.

**Conclusions:**

Among younger adults, CMM was more common in women, whereas among older adults, it was more common in men. Sex also influences patients' histopathological characteristics at diagnosis. Women had better overall survival after adjusting for demographic, pathological, and clinical profiles. The costs of treatment were also lower for women with CMM.

## Introduction

Cutaneous malignant melanoma (CMM) is the deadliest skin cancer,^1,2^ and its worldwide incidence has increased faster than any other malignancy; between 2008 and 2018, CMM incidence rates increased by 44%, with deaths increasing by 32%.^3^ In Italy, between 2008 and 2016, the CMM incidence increased for both sexes (men: +8.8%; women: +7.1%) in all age groups, and CMM is the third most frequent malignancy in both sexes under the age of 50 years.^4,5^

The lifetime risk of CMM was found to be higher for men than for women from middle age onward, whereas the opposite was observed in adolescents and adults up to age 40 years.^6^ Although female cancers are commonly observed to have a significant prognostic advantage, in CMM, this type of a more favorable outcome is considered to be greater than for any other type of malignancy.^7^ This better survival is likely to be attributable largely to more women being diagnosed earlier, with less advanced (and more easily curable) tumors.^8,9^ However, women's prognostic advantage seems to even out in women with more advanced metastatic stages.^5,10^

Few studies have addressed the therapeutic costs of melanoma using real-world data,^11–14^ and even fewer have focused on the stage-specific health care costs.^15,16^ To the best of our knowledge, no studies have compared the costs of health care for melanoma in men versus women.

Based on the data that were recorded in a regional population-based cancer registry, and by linking this information with that of several administrative regional databases, this study aimed to provide a comprehensive/updated overview of the effect of sex on CMM incidence, its clinicopathological profile, and related treatment costs.

## Methods

### Context

The Italian National Health System is a public system that is financed mainly by general taxation, and organized essentially on a regional basis.^17^ Based on national guidelines for the management of CMM, a diagnostic and therapeutic patient care pathway has been adopted in Veneto, a large region in northeastern Italy (with a population of ∼4.9 million), in an effort to ensure the health care system's sustainability, and to reduce inequalities and unwarranted variability in patient management.^18^ In 2017, the regional cancer registry (*Registro Tumori del Veneto, RTV*) set up a high-resolution registry of CMM cases in collaboration with the regional oncology network. Based on patients' clinical records, this registry retrospectively collects details regarding the clinical features and tumor stage at the time of their diagnosis.^19^

### Data and variables

The present retrospective cohort study considered 1,279 cases of CMM who were diagnosed in 2015 in the resident population of the Veneto region, as recorded in the high-resolution regional cancer registry. The following information was available for each patient: (1) tumor site (lower limbs, upper limbs, head, hands/feet, and trunk); (2) ulceration (present vs. absent); (3) histological subtype (malignant melanoma not specified, superficial spreading, nodular, lentigo maligna, acral-lentiginous, desmoplastic, blue nevus, and spitzoid); (4) growth phase (radial or vertical); (5) Breslow thickness (≤0.75; 0.76–1.50; 1.51–3.99; ≥4 mm); (6) number of mitoses (0–2; ≥2 mm^2^); (7) tumor-infiltrating lymphocytes ([TILs], present vs. absent); (8) regression (present vs. absent); and (9) TNM stage at diagnosis (dichotomized as I, II and III, IV).

Through record linkage with the regional mortality registry, vital status was recorded for all cases as of February 29, 2020. The mean follow-up time was 4.3 years.

### Costs

Data on drug prescriptions, use of medical devices, hospital admissions, visits to outpatient clinics and emergency departments, and hospice admissions were obtained from administrative databases.

In particular, the costs were obtained from the reimbursement rates that were established by the Veneto Regional Authority for each procedure or medical service. The following sources of information were used:

- The outpatient database collects information on medical acts and procedures that can be delivered at outpatient facilities under NHS (National Health System) funding, at the rates indicated in the outpatient formulary (NTPA, *Nomenclature Tariffario delle Prestazioni Ambulatoriali*).^20^
- Hospital discharge records include the diagnosis-related group that is associated with each admission, and it is priced at the rate that is indicated in the inpatient formulary (NTPO, *Nomenclature Tariffario delle Prestazioni Ospedaliere*),^21^ which covers all hospital activities (acute or day hospital admissions).
- The regional pharmaceutical distribution database and hospital drug consumption database are used to assess the costs of medical therapies, taking the doses administered into account.
- The emergency department admissions database records the costs of each admission, derived from the rates for all medical acts and procedures that were performed in the emergency departments.
- The medical devices database records the costs that were sustained by the regional authorities to provide medical devices.

Hospice admission costs were also collected by multiplying a regional daily rate by the number of days spent in the hospice.

Each patient was linked through a unique anonymous identification code to all administrative data regarding their hospital admissions, ambulatory care services, drug prescriptions, emergency department visits, medical device usage, and hospice admissions. We considered the overall costs and specific melanoma costs, the latter including only the therapeutic and diagnosis procedures that were specific to the melanoma care pathway,^18^ for up to 1 year of follow-up after CMM was diagnosed.

### Statistical analyses

To establish the melanoma incidence rate, the population that was considered was the mean number of residents in the Veneto Region by age group in 2015. To standardize the sex-specific rates, we considered the world reference population in 2015. Confidence limits of specific rates were calculated, based on a Poisson distribution. Confidence intervals (CIs) of standardized rates were computed according to the formula based on a gamma distribution.

The chi-square test was applied to identify differences in the distribution of categorical histopathological and clinical variables by sex. Fisher's test was only used when there were fewer than five expected cells in a contingency table. The Student's *t*-test or the Mann–Whitney *U* test was used to compare continuous variables, as appropriate (normal distribution was assessed using the Shapiro–Wilk normality test).

The person-year mass was calculated by taking the entry date as the date of diagnosis, and the exit date as the end of the follow-up (February 2020) or death, whichever came first. Cox's regression analysis was used to test the association between sex and survival. We created a first unadjusted model (Model 1) with only sex as a variable, then Model 2 adjusted for age group as well, Model 3 also adjusted for stage distribution at diagnosis, and a fully adjusted model (Model 4) that considered certain histopathological and clinical variables (histological subtype, tumor site, ulceration, mitoses, and TIL).

In the multivariate analysis, we grouped certain histology categories (lentigo maligna, blue nevus, desmoplastic, and spitzoid) in the “Other” modality. Finally, we developed Model 5, in which we checked for any interaction between age and sex, adjusting for previous covariates. When the proportionality assumption for Cox's regression was tested, it had a *p*-value of 0.74 in Model 1, 0.45 in Model 2, 0.41 in Model 3, 0.15 in Model 4, and 0.12 in Model 5.

The R 3.5.2 statistical package was used to record linkage and for all statistical analyses. A *p*-value of <0.05 was considered to be significant. The rates of melanoma between different age groups were compared by calculating the 95% CIs.

### Ethics

Ethical approval for the study was obtained from the Veneto Oncological Institute's Ethics Committee (n. 52/2016). Data analysis was conducted on anonymous aggregated data with no chance of individuals being identifiable.

## Results

The 1,279 patients who were considered in the study were approximately equally distributed by sex (men 53.0%; women 47.0%). Men had a greater mean age at diagnosis at 60.5 years (±15.3 standard deviation [SD]) compared with 56.6 years (±17.0 SD) for women (*p* < 0.001).

The incidence rates differed between men and women, but age was a modifying factor for sex through incidence, as follows: although incidence rates were higher in women than in men among younger patients (<50 years old), though with an overlapping CI, they were significantly higher in men than in women among older adults (>50 years old) (Table 1).

**Table 1.** Incidence Rates of Cutaneous Malignant Melanoma, Overall, and by Sex, Per 100,000 Population: Crude, Standardized (World Standard Population), and by Age Group; Veneto Region, 2015

	Male	Female	Total
Crude rates	28.22	23.8	25.96
0–24 years	1.19 [0.48–2.44]	2.33 [1.24–3.98]	1.74 [1.06–2.69]
25–49 years	20.12 [17.22–23.36]	25.51 [22.21–29.16]	22.79 [20.58–25.18]
50–74 years	47.22 [42.44–52.39]	32.53 [28.68–36.75]	36.69 [36.61–42.95]
75+ years	70.55 [59.46–83.11]	34.84 [28.76–41.82]	48.42 [42.70–54.71]
Standardized rates	18.21 [16.76–19.76]	17.66 [16.15–19.27]	17.91 [16.86–19.01]

The most common primary tumor site for men was the trunk (59.3%), whereas the lower limbs were the most common primary site for women (32.1%), followed by the trunk (31.8%). The trunk location was significantly more frequent in men than in women (*p* < 0.001). Men also had a higher chance (76.3%) of having TIL at diagnosis than women (68.2%) (*p* = 0.004) (Table 2).

**Table 2.** Clinical and Pathological Features of Patients with Cutaneous Malignant Melanoma by Sex

Variable	Total % (*N* = 1,279)	Men % (*N* = 678)	Women % (*N* = 601)	*p*	Variable	Total % (*N* = 1,279)	Men % (*N* = 678)	Women % (*N* = 601)	*p*
Tumor site				**<0.001**	Breslow thickness				0.098
Trunk	46.4 (593)	59.3 (402)	31.8 (191)		≤0.75	54.2 (693)	51.0 (346)	57.7 (347)	
Lower limbs	20.3 (260)	9.9 (67)	32.1 (193)		0.76–1.50	18.4 (236)	18.9 (128)	18.0 (108)	
Head	10.4 (133)	10.8 (73)	10.0 (60)		1.51–3.99	14.0 (179)	16.1 (109)	11.6 (70)	
Upper limbs	15.2 (195)	13.4 (91)	17.3 (104)		≥4	8.3 (106)	8.8 (60)	7.7 (46)	
Hands/feet	4.4 (56)	2.9 (20)	6.0 (36)		Missing	5.1 (65)	5.2 (35)	5.0 (30)	
Missing	3.3 (42)	3.7 (25)	2.8 (17)		Mitotic number				0.747
Ulceration				0.454	0–2	62.4 (798)	61.8 (419)	63.1 (379)	
Absent	78.4 (1,003)	77.1 (523)	79.9 (480)		≥2	19.7 (252)	20.5 (139)	18.8 (113)	
Present	15.8 (202)	17.0 (115)	14.5 (87)		Missing	17.9 (229)	17.7 (120)	18.1 (109)	
Missing	5.8 (74)	5.9 (40)	5.6 (34)		TILs				0.004
Histological subtype				0.053^a^	Absent	14.8 (189)	12.1 (82)	17.8 (107)	
Superficial spreading m.	72.4 (926)	70.9 (481)	74.0 (445)		Present	72.5 (927)	76.3 (517)	68.2 (410)	
Nodular m.	12.4 (159)	14.5 (98)	10.2 (61)		Missing	12.7 (163)	11.6 (79)	14.0 (84)	
Lentigo maligna m.	2.2 (28)	2.2 (15)	2.2 (13)		Tumor regression				0.043
Acral-lentiginous m.	1.9 (25)	1.2 (8)	2.8 (17)		Absent	41.4 (530)	38.2 (259)	45.1 (271)	
Desmoplastic m.	0.3 (4)	0.3 (2)	0.3 (2)		Present	24.8 (317)	26.0 (176)	23.5 (141)	
M. in blue nevus	0.1 (1)	0.0 (0)	0.2 (1)		Missing	33.8 (432)	35.8 (243)	31.4 (189)	
Spitzoid m.	2.2 (28)	1.6 (11)	2.8 (17)		TNM stage				0.058
Malignant melanoma, not otherwise specified	5.8 (74)	6.2 (42)	5.3 (32)		I–II (early stages)	84.4 (1,079)	82.8 (561)	86.2 (518)	
Missing	2.7 (34)	3.1 (21)	2.2 (13)		III–IV (advanced stages)	11.2 (143)	13.1 (89)	9.0 (54)	
Growth phase				0.153					
Missing	22.9 (293)	55.3 (165)	54.2 (128)						

^a^ Fisher's test was applied, otherwise the chi-squared test was used.

TILs, tumor-infiltrating lymphocytes.

**FIG. 1.**
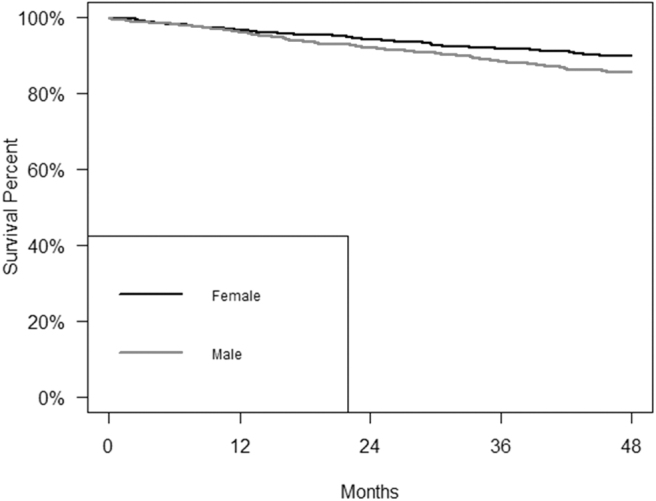
Survival curve of patients with cutaneous malignant melanoma by sex at 48 months. Male—sample, *n* = 678; deaths, *n* = 98; survival rate at 48 months: 85.5%; CI: 82.9–88.2; Female—sample, *n* = 601; deaths, *n* = 61; survival rate at 48 months: 89.9%; CI: 87.5–92.3; log-rank test: *p* = 0.03. CI, confidence interval.

There were no significant sex-related differences in terms of stage at diagnosis, mitoses, Breslow thickness, ulceration, or growth phase. Figure 1 shows the Kaplan–Meier findings. Overall, men had a worse prognosis than women, with a lower overall survival rate at 48 months (85.5 vs. 89.9; *p* = 0.03).

Table 3 gives the results of the Cox regression models for overall survival. Without any adjustment (Model 1), male sex was associated with a higher risk of dying of CMM within 48 months of the diagnosis (hazard ratio [HR]: 1.41; 95% CI: 1.03–1.93). After adjusting for age (Model 2), the survival advantage for women no longer appeared to be significant (HR: 1.28; 95% CI: 0.93–1.75), but after adjusting for both age and stage at diagnosis (Model 3), the risk of dying was significantly lower for women (HR: 1.52; 95% CI: 1.08–2.14). The significance of the association between sex and survival was also evident when histological subtype, tumor site, TIL, mitoses, and ulceration were taken into account (Model 4), (HR: 1.66; 95% CI: 1.05–2.61). In a multivariate analysis (Model 5), the interaction between sex and age was not significant (data not shown).

**Table 3.** Results of the Cox Regression Models for Survival (Only Patients >25 Years Old)

		Model 1			Model 2			Model 3			Model 4		
		HR	95% CI	*p*	HR	95% CI	*p*	HR	95% CI	*p*	HR	95% CI	*p*
Sex (reference: female)	Male	1.41	1.03–1.93	**0.035**	1.28	0.93–1.75	0.133	1.52	1.08–2.14	**0.016**	1.66	1.05–2.61	**0.030**
Age at diagnosis (years) (reference: 25–49 years)	50–74				2.01	1.10–3.67	**0.023**	1.89	0.99–3.60	0.054	3.29	1.26–8.59]	**0.015**
	75+				13.13	7.50–22.98	**<0.001**	8.17	4.42–15.08	**<0.001**	14.83	5.77–38.11	**<0.001**
TNM stage (reference: stage I)	II							4.26	2.70–6.71	**<0.001**	2.33	1.11–4.89	**0.025**
	III							8.65	5.56–13.46	**<0.001**	3.76	1.76–8.05	**<0.001**
	IV							33.06	19.01–57.50	**<0.001**	35.22	12.69–97.71	**<0.001**
Histological subtype (reference: LMM)	ALM										1.70	0.09–31.58	**0.722**
	NM										7.03	0.89–55.85	**0.065**
	SSM										7.57	0.96–59.91	**0.055**
	Malignant melanoma, not specified										3.82	0.41–35.47	**0.239**
	Other										10.94	0.62–193.75	**0.103**
Tumor site (reference: lower limbs)	Upper limbs										0.43	0.20–0.92	**0.030**
	Head/neck										1.06	0.56–2.00	0.852
	Hand and feet										1.29	0.57–2.94	0.545
	Trunk										0.59	0.34–1.04	0.067
TILs (reference: absent)	Yes										1.11	0.68–1.82	0.673
Ulceration (reference: present)	Absent										1.04	0.60–1.81	0.882
Mitoses (reference: 0**–**2)	≥2										2.54	1.32–4.88	**0.005**

Significant values are reported in bold.

ALM, acral lentiginous melanoma; CI, confidence interval; HR, hazard ratio; LMM, lentigo maligna melanoma; NM, nodular melanoma; SSM, superficial spreading melanoma; TILs, tumor-infiltrating lymphocytes.

Table 4 gives the itemized mean health care costs within the first year after diagnosis by sex. Male sex was associated with significantly higher overall costs (€6,888.2 vs. €4,879.0; *p* = 0.002) and melanoma-specific costs (€4,487.5 vs. €3,327.8; *p* = 0.035). In particular, men incurred higher overall costs for hospital admissions (€3,424.4 vs. €2,769.0; *p* = 0.017), which was the greatest item of expenditure for health care in our study population.

**Table 4.** Itemized Mean (and Median) Overall and Melanoma-Specific Health Care Costs in the First Year After Diagnosis by Sex (in Euros)

Overall costs in first year	All sample	Men	Women	Mann–Whitney test
Total overall costs	5,944.1 (2,656.4)	6,888.2 (2,881.5)	4,879.0 (2,441.5)	0.002
Hospital stays	3,116.4 (1,729.7)	3,424.4 (1,729.7)	2,769.0 (1,729.7)	0.017
Outpatient services	1,051.2 (597.4)	1,106.5 (609.8)	988.8 (576.9)	0.251
Direct-distribution pharmaceuticals	1,083.8 (0)	1,388.1 (0)	740.6 (0)	0.083
Other pharmaceuticals	243.2 (91.1)	294.2 (120.9)	185.7 (61.0)	<0.001
Emergency room	50.2 (0)	53.5 (0)	46.6 (0)	0.294
Hospice stays	5.2 (0)	9.9 (0)	0.0 (0)	0.035
Medical devices	394.0 (0)	611.7 (0)	148.4 (0)	0.369

Melanoma-specific costs in first year	All sample	Men	Women	Mann–Whitney test
Total melanoma-specific costs	3,942.5 (2,110.7)	4,487.5 (2,157.5)	3,327.8 (2,057.3)	0.035
Hospital stays	2,135.7 (1,729.7)	2,298.5 (1,729.7)	1,952.0 (1,729.7)	0.109
Outpatient services	901.5 (500.9)	949.2 (501.1)	847.7 (498.6)	0.622
Direct-distribution pharmaceuticals	897.5 (0)	1,228.0 (0)	524.6 (0)	0.244
Other pharmaceuticals	3.1 (0)	3.4 (0)	2.9 (0)	0.591
Emergency room	0.8 (0)	1.0 (0)	0.6 (0)	0.853
Hospice stays	3.9 (0)	7.4 (0)	0.0 (0)	0.060

## Discussion

This population-based study focuses on the relationship between sex and CMM epidemiological and clinicopathological profiles (*e.g.,* incidence, histological phenotypes, and prognosis). The study has also addressed the analysis of treatment-related costs in male compared with female CMM patients.

Among young people, the incidence of CMM was higher in women, but men had a significantly higher incidence in patients older than 50 years; both of these findings are consistent with the results of previous studies that were conducted in fair-skinned populations.^22,23^ The interpretation of these sex-related differences remains controversial.

In young CMM patients, the most credible etiopathogenetic hypothesis mostly involves genetic–environmental interactions that are triggered by occasional ultraviolet radiation exposure in “genetically prone” subjects, whereas later-onset CMM would mostly reflect accumulated lifelong exposure to the sun in less susceptible individuals.^24,25^ Moreover, young women are more likely to be involved in activities/behaviors with a potentially increasing neoplastic risk, including harmful exposure to artificial sun lamps or severe sunburn.^26,27^

Among women, both the CMM stage and the Breslow thickness were lower (although marginally significant); this feature (potentially resulting from women's greater tendency to check their own skin^7,8^) could be consistent with the high frequency of the self-assessment for CMM among women (approximately one in two).^28,29^

The most common primary sites of CMM were the trunk and lower limbs in men and women, respectively. This difference in the primary CMM location has been possibly attributed to clothing styles, with prevalent exposure of the trunk in men and that of the legs/feet in women.^23^ Moreover, the difference in the primary CMM location could, at least partially, provide a reason for the difference in CMM prognosis by sex.^30^ For example, although primary trunk CMMs most frequently metastasize to distant (unpredictable) sites, neoplastic lesions of the lower limb mostly metastasize to regional nodes that are more easily detectable and/or more easily managed surgically.^31^

For the most frequent histological subtypes (covering 1,085 out of 1,279 cases), a borderline-significant frequency of nodular CMM was documented in men, whereas superficial spreading melanoma prevailed in women. An inverse association (by sex) was documented between TILs and tumor regression. TILs are consistently a biological plausibility, and they were more frequently absent in women who also showed a lower frequency of “neoplastic regression.” Conversely, the higher frequency of TIL in males was significantly coupled with a higher frequency of regression.

Thus, these results apparently support a greater tendency for men to have an immunological reaction against CMM, which strongly contrasts with the more favorable CMM outcome that is consistently shown by women. However, the significantly higher frequency of nodular melanoma among men and the clustering of men in the highest Breslow classes may have both contributed to a less favorable prognosis, even if sex still remains a variable that is associated with survival after adjusting for the stage and histotype.^32^

This study associates women with a significantly higher survival rate, and this finding has been confirmed even after adjusting for covariates. However, many studies could not confirm a sex-related advantage after adjusting for stage and Breslow thickness.^7,33–35^ At the molecular level, Gupta et al. documented a significant greater burden of missense mutations (potentially promoting cancer progression) in men, which also suggests a more efficient female-associated immune response.^36,37^

To the best of our knowledge, this is the first large-scale study that specifically quantified the costs of CMM treatment(s) by sex. In the first year after their diagnosis, treatment costs for men were significantly higher, which could potentially be due to both the higher frequency of more advanced CMM stages and the patients' more advanced age, which frequently involves comorbidities-related costs.

This study had some limitations, especially the lack of some variables (*e.g.,* histological categorization of the TIL patterns, and CMM molecular profiling), which could be relevant. Moreover, some variables recorded had many missing data points, which did not allow inclusion of these variables in the multivariate analysis to prevent a sample size reduction.

The main strength(s) of this study is its population-based design, and it provides diagnostic and therapeutic information that was obtained from real-world clinical practice.

In conclusion, this study confirms that the incidence of CMM is higher in male adults >50 years of age, and that some histological features consistently show distinct sex differences. Irrespective of either patient age or the tumor stage, men had a worse overall survival. Finally, men, in their first year after disease diagnosis, generated higher diagnostic and therapeutic-related costs, which was likely due to the more advanced disease at the time of the initial clinical detection.

## Data Availability Statement

The data set generated as part of this study is not publicly accessible, but it is available from the corresponding author (alessandra.buja@unipd.it) upon reasonable request.
